# Elevated Peripheral Frequencies of Th22 Cells: A Novel Potent Participant in Obesity and Type 2 Diabetes

**DOI:** 10.1371/journal.pone.0085770

**Published:** 2014-01-17

**Authors:** Ruxing Zhao, Dongqi Tang, Shounan Yi, Wenjuan Li, Chuanlong Wu, Yiran Lu, Xinguo Hou, Jun Song, Peng Lin, Li Chen, Lei Sun

**Affiliations:** 1 Department of Endocrinology, Qilu Hospital, Shandong University, Jinan, China; 2 Institute of Endocrinology and Metabolism, Shandong University, Jinan, China; 3 Research Center for Cell Therapy, Key Laboratory of Cardiovascular Remodeling and Function Research, Qilu Hospital, Shandong University, Jinan, China; 4 Centre for Transplant and Renal Research, Westmead Millennium Institute, The University of Sydney at Westmead Hospital, Westmead, New South Wales, Australia; Clermont Université, France

## Abstract

**Objective:**

Chronic low-grade inflammation has long been recognized as the central link between obesity and type 2 diabetes (T2D). The novel subset of T helper (Th) cells, Th22, plays an emerging role in chronic inflammation. We investigated the potential association between Th22 and the pathogenesis of obesity and T2D.

**Methods:**

Ninety T2D inpatients (T2D group), 30 healthy participants with BMI ranged from 19 to 23.9 kg/m2 (CTL group) and 30 metabolically healthy obese controls with BMI ≥ 30 kg/m2 (MHO group) were employed in our study. Peripheral frequencies of Th22 and Th1 and Th17 cells were determined by flow cytometry based on their specific cytokine patterns. Cytokine levels in fresh plasma were quantified by ELISA.

**Results:**

Compared to that in CTL group (1.18±0.06%, n = 28), peripheral frequency of Th22 cells was significantly increased in MHO group (1.88±0.10%, n = 30) and in T2D group (2.247±0.10%, n = 89). There was a consistent notable increase in plasma interleukin (IL)-22 of T2D patients [47.56 (30.55–76.89) pg/mL] as compared with that of MHO group [36.65 (29.52–55.70) pg/ml; *P<0.0001] and CTLs [36.33 (31.93–40.62) pg/mL; *P<0.0001]. Furthermore, other than Th1/Th17, previously frequently described participants in obesity and T2D, there was a strong correlation between Th22 frequency and the homeostasis model of assessment for insulin resistance index (*r* = 0.6771, *P<0.0001) and HOMA for β-cell function (*r* = −0.7264, *P<0.0001).

**Conclusions:**

There were increased Th22 frequencies and IL-22 levels in obesity and T2D. Elevated Th22 and IL-22 also aided in the differentiation of MHO from T2D patients. The notable correlation implied that Th22 might play a more determinant role in both insulin resistance and β-cell impairment.

## Introduction

Parallel to type 2 diabetes (T2D), obesity has become a worldwide epidemic concern [1]. Obesity-associated insulin resistance is the key pathological change that leads to T2D. T2D develops when exhausted islet β cells fail to compensate for the increased need for insulin to maintain glucose homeostasis [2]. Evidence from the past decade unequivocally indicates that chronic low-grade inflammation is the major underlying link between these two disorders [3]–[5]. In fact, apart from the long list of metabolic disorders, obesity is frequently reported with an apparent increased prevalence of autoimmune and inflammatory disorders, e.g., asthma [6], rheumatic diseases [7], inflammatory bowel disease [8], and so on. These clinical observations strengthen the case for immunologic imbalance possibly underlying or at least being closely linked to the aggravating metabolic disturbances resulting from nutrition overload. Adipose tissue macrophage accumulation was the first well-described inflammatory participant in obesity, whose recruitment and pro-inflammatory polarization directly contribute to inflammatory status [9], [10]. More recently, Wu et al. [11] and Kintscher et al. [12] extended those observations to cells of adaptive immunity. They suggested that T lymphocyte accumulation occurs in adipose tissue prior to that of macrophages. Moreover, T-cell infiltration accompanied the initiation of insulin resistance and impaired glucose tolerance [11]–[13]. Taken together, these studies indicated that T-cell groups are important regulators of inflammation in both rodent models and obesity and T2D patients.

An appropriate balance between pro-inflammatory and anti-inflammatory T-cell subsets is essential to maintain immune homeostasis and avoid inflammatory diseases. Previous studies in a rodent model of T2D identified an elevation of T helper (Th)1 and Th17 subsets accompanied by a significant decrease of regulatory T cells (Tregs), which may directly trigger activation of innate immunity and thus inflammation, and contribute greatly to insulin resistance [14], [15]. A subsequent study confirmed that there was a skewed pro-inflammatory T-cell compartment in the peripheral blood of T2D patients [16]. In addition, correlation analysis between pro-inflammatory T-cell frequencies and clinical indicators of disease severity has provided fundamental insights into the notion that the imbalance of T-cell subsets is responsible for the development of obesity and T2D [17].

To date, substantial progress in understanding the interactions and counterbalance between helper T-cells in inflammatory status suggests the possibility that new helper T subsets may also play a role in either feedback regulation of immune/inflammatory cell polarization or direct instruction to tissue cells. Recently identified as a distinct lineage of CD4^+^ T cells characterized by their production of interleukin-22 (IL-22), Th22 cells are emergent constitutor in autoimmune and inflammatory diseases [18]. Th22 cells have a specific CCR4^+^ CCR6^+^ CCR10^+^ phenotype and do not express IL-17, CCL20, IL-23R, CD161 (Th17 markers), IL-4 (Th2 marker), or IFN-γ (Th1 marker) [19]. Aryl hydrocarbon receptor is the key transcription factor determining the lineage commitment of Th22 cells from naïve CD4^+^ T (Th0) cells, whose activation can be positively regulated by IL-6 and TNF-α [18], [20]. It has been proven that IL-22, a cytokine predominantly secreted by Th22 cells, is upregulated in many chronic inflammatory diseases [21]. Accumulating evidence supports an important and complicated role for Th22 cells in chronic inflammation and autoimmune diseases such as psoriasis, rheumatoid arthritis, ankylosing spondylitis, immune thrombocytopenia, Graves' diseases [22]–[25], and the like. Although the skewed pro-inflammatory phenotype of CD4^+^ T subsets that include Th1, Th17, and Treg cells is frequently reported, there is no study at present on the frequencies and function status of Th22 cells in obesity and T2D patients. In the present study, we determined the frequencies of peripheral Th22, Th1, and Th17 cells and plasma levels of the cytokines IL-22, IFN-γ, IL-17, IL-6, and TNF-α. The relevance between Th22 cells and clinical patient data was evaluated.

## Research Design and Methods

### Subjects and ethics statement

Enrollment took place between April 2012 and January 2013 in Qilu Hospital, Shandong University, China. Subjects were assigned to three groups. The T2D group comprised clinically definite T2D inpatients from the Qilu Hospital Department of Endocrinology. Healthy (CTL; BMI: 19–23.9 kg/m^2^) and metabolically healthy obese (MHO; BMI ≥ 28 kg/m^2^) control groups were formed with volunteers from the community and hospital staff. Subjects in the MHO group had increased weight but normal glucose tolerance and lipid profiles. A detailed clinical record was registered for each subject, including history of disease and physical and laboratory examinations. Exclusion criteria were any signs of autoimmune disorders, recent infections, tumors, fever of any cause, and significantly elevated erythrocyte sedimentation rate. Participants did not receive immunosuppressive or immunomodulatory drugs for at least three months during sampling. The Medical Ethical Committee of Qilu Hospital approved this study. A signed consent form was obtained from each participant. Baseline clinical parameters are summarized in Table 1.

**Table 1:** Demographic characteristics of participants.

	CTL	MHO	T2D
Number	30	30	90
BMI (kg/m^2^)	22.19±0.24	30.37±0.45	24.89±0.47
Age (year)	44.43±1.75	42.47±2.18	54.36±1.18
Sex (female/male)	14/16	13/17	43/47
FBG (mM)	5.19±0.08	5.29±0.08	10.63±0.28
2 h BG (mM)	5.81±0.23	5.98±0.13	12.25±0.40
FINS (IU/mL)	7.44±0.45	11.54±0.77	11.34±0.79
2 h INS (IU/mL)	n.a.	43.96±1.83	37.14±2.27
ICA/GAD/IAA	n.a.	n.a.	Negative
Duration of illness (year)	n.a.	n.a.	6.14±1.63

FBG: Fasting blood glucose; 2 h BG: blood glucose level 2 h after a meal;

FINS: fasting insulin level; 2 h INS: insulin level 2 h after a meal;

ICA: islet cell antibodies;

GAD: autoantibody for glutamate decarboxylase;

IAA: insulin antibodies

n.a: not applicable

### Sample preparation

Fasting peripheral blood samples were obtained from each donor between 7 and 9 a.m. All samples were processed within 2 h after collection. Blood (6 mL) for further incubation or isolation was drawn into BD Vacutainer® Heparin (sodium) Tubes (BD, Franklin Lakes NJ, USA). Heparinized peripheral whole blood (400 µL) diluted with an equal volume of RPMI 1640 medium containing 50 ng/mL phorbol myristate acetate (PMA), 2 µg/mL ionomycin, and 3.4 µg/mL monensin (Sigma-Aldrich, St. Louis, MO, USA) was incubated for 4.5 h at 37°C in 5% CO_2_. PMA and ionomycin function synergistically as T-cell activators through non–antigen-specific activation of PKC and by increasing intracellular Ca^2+^, mimicking signals induced by the T-cell receptor complex. Monensin, a protein transport inhibitor, leads to intracellular cytokine accumulation, thus facilitating subsequent detection by flow cytometric analysis.

### Cell staining and flow cytometric analysis

Stimulated whole blood was stained for flow cytometric study to identify cytokine-producing cells. After incubation, 100 µL blood was stained with PerCP-Cy5.5–conjugated anti-human CD4 monoclonal antibodies (clone: OKT4, Cat.: 85-45-0048-42) at room temperature for 20 min. The sample was then treated with an equal volume of Fix-Perm reagent A for 15 min, and then washed with pre-cooled washing buffer. Subsequently, Fix-Perm reagent B was added for further permeabilization for intracellular staining and erythrocyte lysis. The sample was incubated for 20 min together with PE-conjugated anti–IFN-γ monoclonal antibodies (clone: 4S.B3, Cat.: 85-11-7319-82), FITC-conjugated anti–IL-17A monoclonal antibodies (clone: eBio64D, Cat.: 85-11-7179-42), and eFluro680-conjugated anti–IL-22 monoclonal antibodies (clone: 22URTI, Cat.: 85-50-7229-42). Isotope controls were used for each staining procedure as negative controls and for fluorescence compensation. All antibodies were from eBioscience, San Diego, CA, USA. Fix-Perm reagents were from Invitrogen (Carlsbad, CA, USA). All samples were washed and collected using BD Accuri C6 Flow Cytometer. Data were analyzed with FlowJo 7.6.

### Quantification of plasma cytokines by using enzyme-linked immunoassay

The plasma levels of Th1/Th17/Th22-predominantly secreted cytokines IFN-γ, IL-22, IL-17A, and the cytokines previously reported as major drivers of Th22 commitment from Th0 cells, i.e., IL-6 and TNF-α, were determined with a quantitative sandwich ELISA according to the manufacturer's recommendations (Multisciences, CN). The minimum detectable concentration of IFN-γ, IL-22, IL-17A, IL-6 and TNF-α is 0.30, 2.56, 0.55, 0.37, 0.42 pg/ml respectively.

### Statistical analysis

Results are expressed as mean ± SEM or median (range). Differences in Th22, Th17, and Th1 cell frequencies; plasma cytokine levels; and expression of specific transcription factors in PBMC among T2D, MHO patients, and CTLs were determined by using ANOVA, and differences between two groups were determined by using the Newman–Keuls multiple comparison test (q test). Data not normally distributed were analyzed with the Kruskal–Wallis test (H test) and Dunn test. Odds ratio was calculated in a 2×2 arranged table with Fisher's exact test. The Pearson or Spearman correlation test was used for correlation analysis depending on data distribution. Partial correlation analysis was conducted to role out confounding factors. SPSS 18.0 or GraphPad Prism 5.0 were used to perform all tests and generate values. A *P*-value of less than 0.05 was considered statistically significant.

## Results

### Circulating Th22 cells increased in tandem with Th1/Th17 cell frequencies in MHO and T2D patients

**Figure 1:**
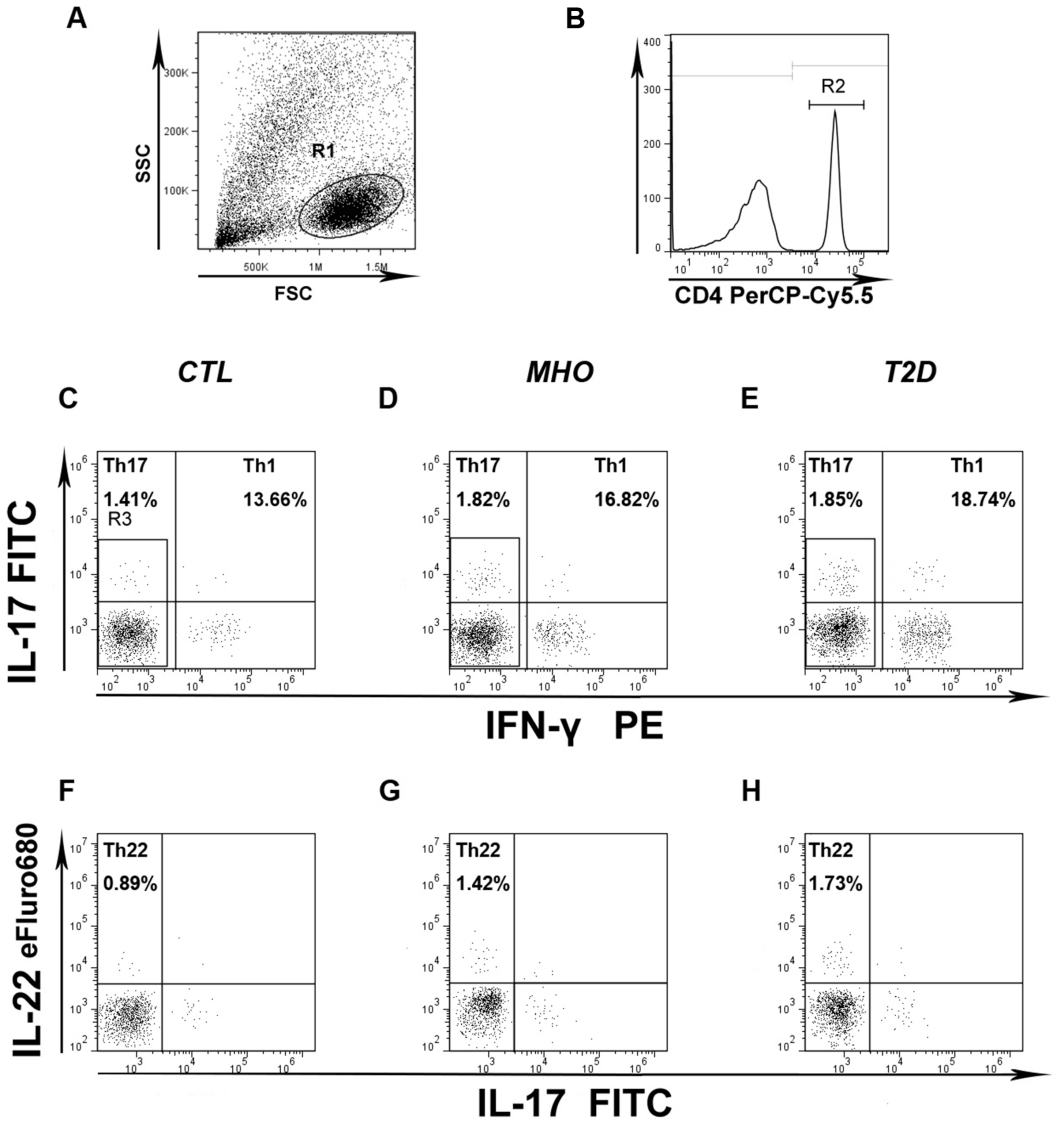
Gating strategy and representative dot plots for flow cytometry analysis. CD4^+^ T cells (B) were gated from lymphocytes (A) for further phenotyping. (C, D, E) Representative dot plots of peripheral Th1 (CD4^+^ IFNγ^+^; UR plus LR quadrant) and Th17 frequencies (CD4^+^ IFNγ^−^IL-17^+^; UL quadrant) from CTLs, MHO, and T2D patients. (F, G, H) Representative FCM dot plots of peripheral Th22 cells (CD4^+^ IFNγ^−^IL-17^−^IL-22^+^; UL quadrant) from CTLs, MHO, and T2D patients. Numbers in each plot denote the percentages of the indicated helper T subset in the total CD4^+^ T-cell population.

**Figure 2:**
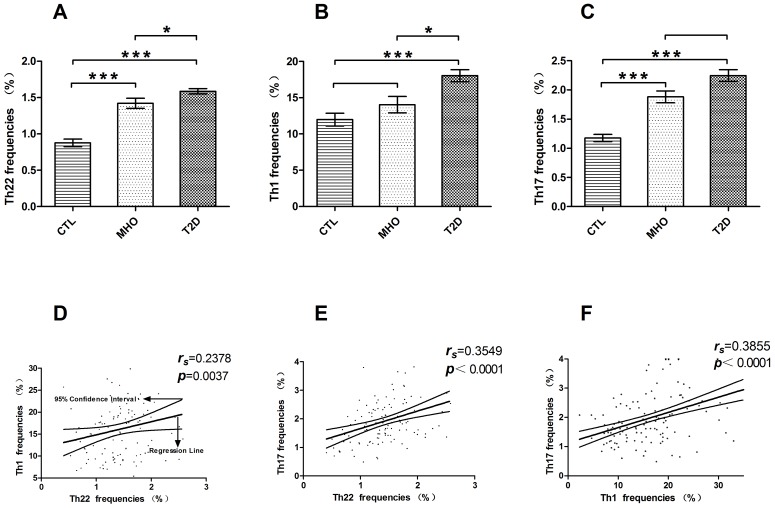
Increased percentages of Th22 cells in peripheral blood from MHO and T2D patients and their correlation with Th1/Th17 frequencies. Circulating percentages of Th22, Th1, and Th17 cells from CTLs, MHO, and T2D patients. (A) Significantly increased percentages of Th22 cells in MHO (1.88±0.10%) and T2D patients (2.247±0.10%) as compared to CTLs (1.18±0.06%) (*P<0.0001). Increased percentages of Th22 cells were also found in MHO patients as compared to T2D patients (*P<0.05). (B) Significant increases in Th1 cells were observed in T2D patients (median, 17.73%; range, 4.652%–42.52%) as compared to CTLs (median, 11.17%; range, 4.724%–25.70%; *P<0.0001) and MHO patients (median, 15.23%; range, 2.297%–27.56%; *P<0.05). There was no significant difference between MHO patients and CTLs. (C) Significantly elevated percentages of Th17 cells were found in T2D (median, 2.08%; range, 0.48%–5.25%; *P<0.0001) and MHO patients (median, 1.815%; range, 1.10%–3.21%; *P<0.0001) as compared to CTLs (median, 1.18%; range, 0.60%–1.80%). There was no significant difference between MHO and T2D patients. (D, E, F) Correlation between the percentages of Th22, Th1, and Th17 cells from all blood donors. There was a positive correlation among all three subsets of helper T cells. Bars represent SEM. Numbers in each figure denote Spearman *r* and *P* values.

The peripheral frequencies of Th22, Th1, and Th17 cells were analyzed based on their specific cytokine patterns after 5-h stimulation in the presence of PMA, ionomycin, and monensin. The gating procedure and typical dot plots in defining the expression of circulating Th22 (CD4^+^ IFNγ^−^IL-17^−^IL-22^+^), Th1 (CD4^+^ IFNγ^+^), and Th17 (CD4^+^ IFNγ^−^IL-17^+^) cells are depicted in Figure 1. Compared to CTLs (1.18±0.06%, n = 28), the frequency of peripheral Th22 cells was significantly increased (1.59 folds) in MHO (1.88±0.10%, n = 30; *P<0.0001) and (1.90 folds) T2D patients (2.247±0.10%, n = 89; *P<0.0001). Even higher percentages (1.31 folds) of Th22 cells were observed in T2D compared to MHO patients (*P<0.05) (Fig. 2A).

To further evaluate the increased risk associated with elevated Th22 for health people to develp obesity and for obese people to develop diabetes respectively, the odds ratio was calculated in a 2×2 table (with continuity correction of 0.5 to each cell when needed) (see Table 2 and Table 3 respectively). The median 1.40% was selected as the threshold of normal and elevated Th22 frequency. In the sub-cohort of metabolically health subjects(n = 58), the odds ratio was up to 54.47 (*P<0.0001). And in obese people who had an elevated Th22, diabetes might occur 8.636 times as often as in those with normal Th22 frequency(*P = 0.0148), indicating an potential value of Th22 in predicating obese outcomes. However, since Th22 frequency is a continuous variable and there is currently no wide-accepted categorical threshold, we could only make artificial definition according to our data. Moreover, statistical values should be for reference only since the cases of participants in our study did not represent disease prevalence in the natural population.

**Table 2:** Peripheral Th22 frequency and prevalence of obesity in the subset of metabolically healthy participants.

Group	Health	Obesity	Total
Normal	28	19	47
Elevated	0	11	11
Total	28	30	58

**Table 3:** Peripheral Th22 frequency and prevalence of T2D in the subset of obese participants.

Group	MHO	T2D	Total
Normal	19	2	21
Elevated	11	10	21
Total	30	12	42

Besides, there was also a significant increase of Th1 frequency in T2D patients (median, 9.06%; range, 6.01%–14.02%; n = 89) as compared with MHO patients (median, 9.06%; range, 6.01%–14.02%; n = 30) (*P<0.0001) and CTLs (median, 9.06%; range, 6.01%–14.02%; n = 28) (Fig. 2B). No significant difference was found in Th1 frequency between MHO patients and CTLs. Consistent with a previous study, Th17 cells were increased in the peripheral blood of MHO (median, 9.06%; range, 6.01%–14.02%; n = 30) and T2D patients (median, 9.06%; range, 6.01%–14.02%; n = 89) as compared with CTLs (median, 9.06%; range, 6.01%–14.02%; n = 28) (*P<0.0001). However, our findings failed to demonstrate a significant difference in Th17 frequency between T2D and MHO patients (Fig. 2C).

As preliminary exploration of the interactions between the three pro-inflammatory helper T subsets, inter-subset correlation was evaluated. There was a significantly positive correlation between the frequencies of peripheral Th22 and Th1 cells (*r* = 0.2378, *P = 0.0037, n = 147, Spearman analysis; Fig. 2D), Th22 and Th17 cells (*r* = 0.3549, *P<0.0001, n = 147, Spearman analysis; Fig. 2E), and Th17 and Th1 cells (*r* = 0.4387, *P<0.0001, n = 147, Spearman analysis; Fig. 2F).

### Plasma levels of IL-22, IFNγ, and IL-17 were elevated in non-stimulated peripheral blood from MHO and T2D patients

**Figure 3:**
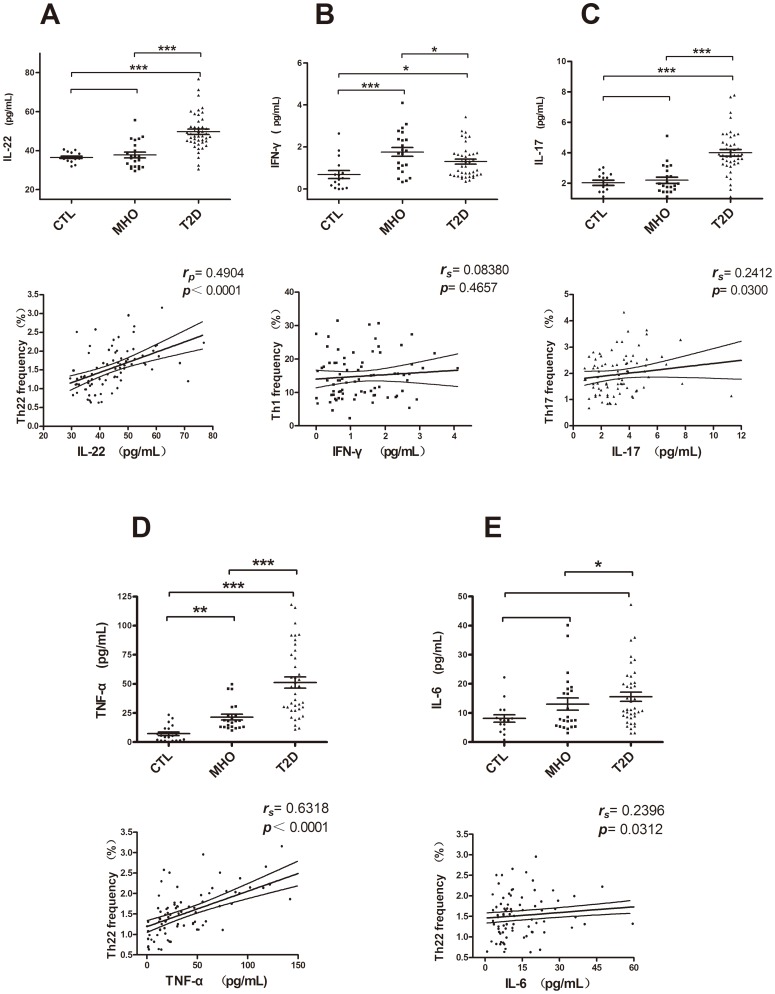
Concentrations of related plasma cytokines in non-stimulated peripheral blood. Concentrations of IL-22, IFN-γ, IL-17, TNF-α, and IL-6 in plasma from CTLs, MHO, and T2D patients. (A) Significantly higher concentrations of IL-22 were observed in T2D patients (median, 47.56 pg/mL; range, 30.55–76.89 pg/mL) as compared to CTLs (median, 36.33 pg/mL; range, 31.93–40.62 pg/mL; *P<0.0001) and MHO patients (median, 36.65 pg/mL; range, 29.52–55.70 pg/mL; *P<0.0001). There was no significant difference in IL-22 between MHO patients and CTLs (*P*>0.05). (B) Significantly higher concentrations of IFN-γ were found in MHO (median, 1.76 pg/mL; range, 0.33–4.10 pg/mL; *P<0.0001) and T2D patients (median, 1.08 pg/mL; range, 0.36–3.43 pg/mL; *P<0.05) as compared to CTLs (median, 0.48 pg/mL; range, lower limit to 2.64 pg/mL). No significant difference was found between MHO and T2D patients. (C) Significantly higher concentrations of IL-17 were found in T2D patients (median, 3.84 pg/mL; range, 0.81–7.78 pg/mL) as compared to CTLs (median, 2.06 pg/mL; range, 0.81–3.04 pg/mL; *P<0.0001) and MHO patients (median, 1.91 pg/mL; range, 0.89–5.11 pg/mL; *P<0.0001). There was no significant difference between MHO patients and CTLs (*P*>0.05). (D) Significantly elevated TNF-α levels were found in MHO (median, 18.15 pg/mL; range, 10.08–49.81 pg/mL; *P<0.01) and T2D patients (median, 46.98 pg/mL; range, 11.07–118.1 pg/mL; *P<0.0001) as compared with CTLs (median, 5.28 pg/ml; range, lower limit to 23.36 pg/mL). Higher concentrations of TNF-α were also observed in T2D patients compared with MHO patients (*P<0.0001). (E) Significantly elevated IL-6 levels were observed in T2D patients (median, 11.21 pg/mL; range, 3.09–47.24 pg/mL) as compared to CTLs (median, 7.53 pg/mL; range, 0.79–22.24 pg/mL; *P<0.05). There was no significant difference between MHO patients (median, 7.85 pg/mL; range, 3.16–40.12 pg/mL) and CTLs and T2D patients (*P*>0.05). Correlation analyses between the indicated helper T-cell frequencies and corresponding plasma cytokine levels from each sub-cohort. There was a positive correlation between Th22 frequency and IL-22 concentration (A) and between Th17 and IL-17 (C). There was also a significant positive correlation between Th22 frequency and plasma TNF-α (D) (*P<0.0001) and IL-6 (E) (*P = 0.0312) levels. Bars represent SEM. Numbers in each figure denote Spearman or Pearson *r* and *P* values, respectively.

To test the hyperactivity of the pro-inflammatory CD4^+^ helper T subsets in non-stimulated fresh peripheral blood from MHO (n = 22) and T2D patients (n = 45), plasma levels of the corresponding predominant cytokines IL-22, IFN-γ, and IL-17 were measured by using ELISA. As shown in Figure 3A, there was a notable increase in plasma IL-22 from T2D patients (median, 47.56 pg/mL; range, 30.55–76.89 pg/mL) as compared with MHO patients (median, 36.65 pg/mL; range, 29.52–55.70 pg/mL; *P<0.0001) and CTLs (median, 36.33 pg/mL; range, 31.93–40.62 pg/mL; n = 16; *P<0.0001). However, there was no significant difference between MHO and CTLs (*P*>0.05). Similarly, the concentrations of plasma IL-17 from our blood donors were consistent (Fig. 3B). There were significantly higher concentrations of IL-17 in T2D patients (median, 3.84 pg/mL; range, 0.81–7.78 pg/mL) as compared with CTLs (median, 2.06 pg/mL; range, 0.81–3.04 pg/mL; *P<0.0001) and MHO patients (median, 1.91 pg/mL; range, 0.89–5.11 pg/mL; *P<0.0001). There was no significant difference between MHO patients and CTLs (*P*>0.05). There were also significantly higher concentrations of IFN-γ in MHO (median, 1.76 pg/mL; range, 0.33–4.10 pg/mL; *P<0.0001) and T2D patients (median, 1.08 pg/mL; range, 0.36–3.43 pg/mL; *P<0.05) as compared to CTLs (median, 0.48 pg/mL; range, lower limit to 2.64 pg/mL). No significant difference was found between MHO and T2D patients (*P*>0.05) (Fig. 3C).

For further analysis of the correlation between elevated helper T-cell frequencies and their predominantly secreted cytokines, a correlation study was performed in all plasma donor sub-cohorts (n = 81). As shown in Figure 3A and 3C, there was a significant positive correlation between Th22 frequency and IL-22 concentration (*r* = 0.4904, *P<0.0001, Pearson analysis) as well as between Th17 frequency and IL-17 concentration (*r* = 0.2412, *P = 0.0300, Spearman analysis). However, there was no statistical correlation between peripheral Th1 cells and non-stimulated circulating levels of IFN-γ (*r* = 0.0838, *P* = 0.4657, Spearman analysis) (Fig. 3B).

### Elevated plasma levels of IL-6 and TNF-α in MHO and T2D patients promoted Th22 polarization

TNF-α and IL-6 have been reported as major drivers of Th22 polarization[18], [20]. In the present study, we also quantified plasma concentrations of TNF-α and IL-6 by using ELISA. Concordant with previous studies, there were significantly elevated TNF-α levels in MHO (median, 18.15 pg/mL; range, 10.08–49.81 pg/mL; *P<0.01) and T2D patients (median, 46.98 pg/mL; range, 11.07–118.1 pg/mL; *P<0.0001) as compared with CTLs (median, 5.28 pg/mL; range, lower limit to 23.36 pg/mL). There were also significantly higher concentrations of TNF-α in T2D patients as compared to MHO patients (*P<0.0001) (Fig. 3D). While significantly elevated IL-6 levels were observed in T2D patients (median, 11.21 pg/mL; range, 3.09–47.24 pg/mL) as compared to CTLs (median, 7.53 pg/mL; range, 0.79–22.24 pg/mL; *P<0.05), we found no significant difference between MHO patients (median, 7.85 pg/mL; range, 3.16–40.12 pg/mL) and CTLs as well as T2D patients (*P*>0.05) (Fig. 3E). Moreover, correlation analysis revealed the significant relevance of TNF-α (*r* = 0.6318, *P<0.0001, Spearman analysis) and IL-6 levels (r = 0.2396, *P = 0.0312, Spearman analysis) to Th22 frequency.

### Elevated Th22 frequency was bound with insulin resistance in MHO and newly diagnosed T2D patients

**Figure 4:**
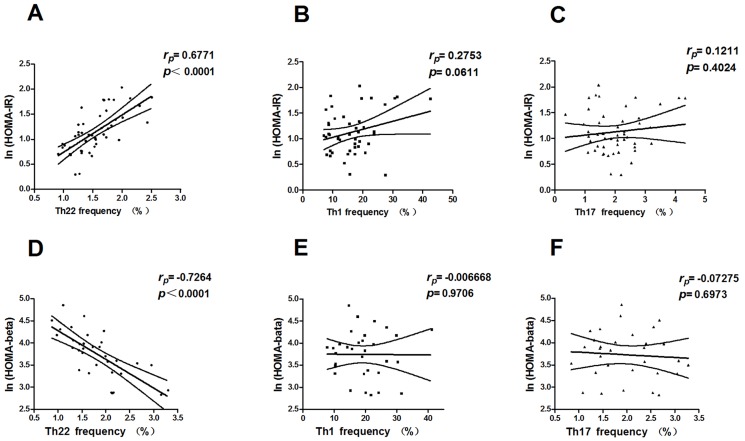
Correlation of peripheral helper T subset percentages with HOMA-IR and HOMA-β. There was a significantly positive correlation of Th22 frequency with ln(HOMA-IR) in MHO and newly diagnosed T2D patients (*P<0.0001) (A) and with ln(HOMA-β) (*P<0.0001) (D) in later-stage T2D patients. (B, C, E, F) The correlation of ln(HOMA-IR) or ln(HOMA-β) value with Th1 or Th17 frequency was not statistically significant. Numbers in each figure denote Pearson *r* and *P* values, respectively.

As our study revealed an expanded Th22 population in patients with MHO and T2D, we further studied the potential role of this hyperactive phenotype in the development of impaired insulin sensitivity. The homeostasis model of assessment for insulin resistance index (HOMA-IR) has been widely accepted as a good indicator of insulin resistance, especially in patients with comparatively well preserved islet β-cell functions, e.g., MHO patients, prediabetic individuals (impaired glucose tolerance or impaired fasting glucose), and some early-diagnosed T2D patients [26]. Its natural logarithmic value, ln(HOMA-IR), is an even more reliable parameter. In our study, correlation analysis of Th22 frequency and the ln(HOMA-IR) value in a sub-cohort (n = 50) of MHO and newly diagnosed T2D patients revealed the notable relevance of the expanded Th22 population to increased insulin resistance (*r* = 0.6771, *P<0.0001, Pearson analysis) (Fig. 4A). However, neither Th1 (Fig. 4B) nor Th17 cells (Fig. 4C), previously described pro-inflammatory CD4^+^ helper T subsets, had a significant correlation with the ln(HOMA-IR) index. Further, partial correlation analysis in female (n = 22) and male (n = 28) was conducted with the confounding effects of age and sex removed. Significant correlationship between Th22 frequencies and ln(HOMA-IR) values was still present in both male (*r* = 0.713, *P<0.0001) and female (*r* = 0.487, *P = 0.03) subsets of participants.

### Hyperactive Th22 phenotype negatively correlates with residual islet β-cell function in late-stage T2D

As an even more significant expansion of the Th22 population and elevated IL-22 secretion was observed in T2D patients, we investigated the idea of whether the hyperactive Th22 phenotype correlated with the decompensation of β-cell function. The HOMA for β-cell function (HOMA-β) was introduced as a parameter of the residual basal insulin-secreting function of β cells in patients with later-stage T2D [26]. There was an obvious β-cell function loss in this sub-cohort of T2D patients (n = 33), characterized by exhausted insulin secretion. Our findings revealed a remarkable negative correlation between Th22 frequencies and ln(HOMA-β) values (*r* = −0.7264, *P<0.0001, Pearson analysis) (Fig. 4D). There was no significant correlation between ln(HOMA-β) values and Th1 or Th17 frequencies (Fig. 4E, 4F). Correlationship between Th22 frequencies and ln(HOMA-β) values remained statistically significant with age controlled by partial correlation analysis in male (*r* = 0.607, *P = 0.013, n = 17) and female (*r* = 0.850, *P<0.0001, n = 16) subsets of participants respectively.

## Discussion

Along with T2D, obesity shares a common pathological process of insulin resistance. It has been proven that insulin resistance is the core initiating and persisting factor in T2D and obesity-associated metabolic syndrome, while the steady loss of β-cell function results in the aggravation of hyperglycemia and development of diabetic complications [2]. In the past two decades, obesity and T2D have been linked to chronic low-grade systemic inflammation, which is postulated to be causal throughout the development of insulin resistance and progression to diabetic complications [3]. Obesity is continually reported with predisposition to autoimmune disorders [6]–[8]. Moreover, a growing body of evidence published in recent years suggests a substantially increased risk for developing malignancies in patients with T2D [27]. These epidemiologic clues also indicate a plausible link from metabolic pressures to immunologic disturbance and inflammation. However, the specific immunologic sensors triggered in response to metabolic dysfunction to produce a state of inflammation have not been identified. Recent progress in the interaction between immune cell subsets, particularly extension of the knowledge on reciprocal regulation and counterbalance between helper T-cell subsets, sheds some light on the puzzle. Previous studies in rodent obesity/T2D models and patients identified a skewed pro-inflammatory T-cell compartment characterized by elevated Th1 and Th17 cells and decreased Treg cells [11], [12], [15]–[17]. Correlation analysis between pro-inflammatory T-cell frequencies and metabolic indicators also implied that the imbalance between T-cell subsets is responsible for the development of obesity and T2D in humans [17].

Compared with the well-known Th1, Th2, Th17, and Treg subsets, Th22 is a newly identified helper T-cell subset with a specific phenotype and distinct function. Th22 cells possess a specific cytokine profile that includes IL-22 and TNF-α, both frequently reported to play important roles in chronic inflammation and tumorigenesis [28], [29]. Unlike other cytokines, IL-22 receptors are absent on immune cells, being restricted to tissues instead, thus providing signal directionality from the immune system to tissues [21]. IL-22 regulates cell activity via JAK-STAT3 pathway[21], [30]. Though the direct effects of IL-22 on metabolism were poorly reported, there were ermerging studies involving the JAK-STAT3 regulation on metabolism. Chronic activatated JAK-STAT3 pathway has been summarized to contribute to obesity [31] and peripheral insulin resistance[32], [33] as well as leptin resistance[33].On the other hand, TNF-α is one of the best described causative factors in obesity-associated insulin resistance and the pathogenesis of T2D [34]. This raises the possibility that Th22 cells might play an independent role in the development of obesity and T2D.

To study whether the Th22 subset is involved in the process of obesity and T2D, the percentages of peripheral Th22 cells in each participant were determined by using flow cytometry. Our study first identified a notably expanded peripheral Th22 population (defined as CD4^+^ IFN-γ^−^IL-17^−^IL-22^+^) in both obesity and T2D patients as compared with healthy donors. The increased Th22 population could even aid in distinguishing MHO patients from T2D patients. The increase in Th22 cells was more significant in comparison with Th1 and Th17 cells, the previously described pro-inflammatory subsets in obesity and T2D. There were also consistently increased levels of IL-22 in both MHO and T2D patients, which was in concert with the previously described inflammatory cytokines. These results indicate that Th22 cells might play a greater role in the immunologic disturbance contributing to disease progression.

As our study revealed an expanded Th22 population in patients with MHO and T2D, we evaluated the potential role of Th22 cells in disease progression. The remarkable positive correlation of ln(HOMA-IR) values with Th22 but not Th1 or Th17 frequencies indicates that Th22 cells may contribute greatly to insulin resistance in both MHO and T2D patients. As we observed an even more hyperactive Th22 phenotype in T2D patients, we further investigated whether Th22 cells exerted a negative effect on islet β-cell function. Again, there was a strong negative correlation of Th22 cells with the clinical indicator of basal β-cell function, ln(HOMA-β). This finding preliminarily demonstrates a causative or even determinant role of Th22 cells in the progressive loss of β-cell function. However, the underlying mechanisms have yet to be discussed.

To explore the sources of the expanded Th22 population, we determined the plasma levels of TNF-α and IL-6, which have been identified as major drivers of Th22 lineage commitment [20]. Consistent with previous studies [35], [36], plasma TNF-α levels were elevated in patients with MHO and T2D. In T2D patients, there were even higher TNF-α concentrations accompanied by increased IL-6 levels. In a positive feedback manner, Th22 cells are also a major source of TNF-α. Interestingly, TNF-α has long been established as an important mediator of insulin resistance [34], [36]–[39] and β-cell apoptosis [40]. However, despite its pro-diabetic role being clarified, neutralizing TNF-α in T2D patients was insufficient for reversing the metabolic disturbance [34]. Moreover, without affecting insulin receptor-mediated signal transduction, TNF-α can induce insulin resistance in adipocytes, characterized by loss of insulin receptor substrate-1 and GLUT4 expression [37]. Taken altogether, we hypothesize that apart from its direct influence on adipose tissue and β cells, TNF-α might contribute to the pathogenesis of obesity and T2D in a Th22-dependent manner. Once over-activated by TNF-α and IL-6, Th22 cells exacerbate insulin resistance and aggravate β-cell injuries, thus promoting disease progression.

The study of how metabolic pressures affect the modulation of immune homeostasis [41] and how immune imbalance leads to chronic inflammation in return [42] are now subjects of intensive investigation. While the basic mechanisms governing inflammatory status in the context of metabolic and immunologic disturbance remain to be elucidated, clinical observations provide more clues for further exploration that might be indispensable. Collectively, we identified for the first time a significant elevation of Th22 frequency from the peripheral blood of MHO and T2D patients. An even more hyperactive phenotype of Th22 cells was identified in T2D patients, which renders it of potential value in predicating obese outcomes and evaluating the intervention effect in individuals. Preliminarily, our data suggest that Th22 cells might play a dual role in both the development of insulin resistance and β-cell dysfunction. However, as a single-centered cross-sectional research, our study was exploratory and could not confirm the cause-effect relationship. Further prospective studies with larger sample sizes, animal models as well as in vitro experiments are warranted to validate the potentially causative role of increased Th22 in disease progression of obesity and type 2 diabetes.
